# Automated, Point-of-Care mobile flow cytometry: Bringing the laboratory to the sample

**DOI:** 10.1016/j.heliyon.2024.e28883

**Published:** 2024-04-03

**Authors:** B.N. Jukema, T.C. Pelgrim, M. Spoelder, C.C.W.G. Bongers, M.T.E. Hopman, K. Smit, M.H. Rijk, R.P. Venekamp, N. Vrisekoop, L. Koenderman

**Affiliations:** a Department of Respiratory Medicine, University Medical Center Utrecht, Utrecht University, Utrecht, Netherlands; b Center for Translational Immunology, University Medical Center Utrecht, Utrecht University, Utrecht, Netherlands; c Department of Medical BioSciences, Radboud University Medical Center, Nijmegen, Netherlands; d Department of General Practice and Nursing Science, Julius Center for Health Sciences and Primary Care, University Medical Center Utrecht, Utrecht University, Utrecht, Netherlands

**Keywords:** Mobile flow cytometry, Point-of-Care, Systemic inflammation, Innate immune activation, Neutrophil, Monocyte, Monocyte subsets, Eosinophil, Near patient, First line care

## Abstract

**Background:**

Innate effector cells are very responsive to infectious and inflammatory cues found in damaged and inflamed tissues. Their activation is a potential target to assess the state of the immune system. Unfortunately, these cells are very susceptible for ex-vivo activation, hampering accurate interpretation of flow cytometry data. Whether a brief window exists before ex-vivo activation starts to occur is currently unknown.

**Aims:**

1) This study extensively investigated ex-vivo activation of innate effector cells over time. 2) We tested the feasibility of applying a mobile, automated, flow cytometry laboratory for out-of-hospital Point-of-Care analyses to minimize ex-vivo activation bias.

**Methods:**

1) Ex-vivo neutrophil, eosinophil and monocyte activation in a blood collection tube over time and the reactivity to a formyl-peptide was investigated in a healthy cohort. 2) To facilitate fast, out-of-hospital analysis, application of the mobile flow cytometry was tested by placing an automated flow cytometer into a van. The stability of the setup was assessed by repetitively measuring laser alignment and fluorescence verification beads.

**Findings:**

1) Immediately after venipuncture activation marker expression on neutrophils, eosinophils and monocyte subsets started to change in a time-dependent manner. 2) The mobile flow cytometry laboratory travelled over 3000 km, performing measurements at 19 locations with a median single-person-set-up time of 14 min. The laser alignment and fluorescence were stable during all experiments.

**Conclusions:**

Accurate flow data of innate immune cells are only obtained when ex-vivo activation is kept to minimum. The use of a mobile, fast, automated, flow cytometry laboratory for out-of-hospital Point-of-Care analyses provides new investigational and diagnostic possibilities outside major hospital flow cytometry laboratories.

## 1 Introduction

Neutrophils, monocytes and eosinophils are the main effector (blood) cells of the innate immune system. These cells are the first to respond to infectious (e.g. microbe-associated molecular patterns) and other inflammatory cues (e.g. damage-associated molecular patterns; DAMP's and cyto-/chemokines) which are found in damaged and/or inflamed tissues [1]. They have different functions necessary for an array of responses ranging from elimination of pathogens to correct repair of damaged tissues. The cells are also implicated in immune regulation and are involved in the pathogenesis of chronic inflammatory diseases [[2], [3], [4]]. This makes the immunophenotyping of the cells an interesting way to study the ‘state’ of the immune system in both the acute and chronic inflammatory and/or infectious setting.

The high sensitivity of these ‘first-responder’ cells for environmental cues is also reflected by their responsiveness to ex-vivo manipulation, which makes these cells challenging to study. We have recently demonstrated that neutrophils, monocytes and eosinophils get activated quickly in a blood collection tube at room temperature: the activation markers were already significantly increased when the analysis of these cells was postponed more than 1 h after venipuncture [5]. It is still unknown whether ex-vivo activation of these cells starts immediately after venipuncture, or whether there is a small window of opportunity before ex-vivo activation takes place. Moreover, the effect of ex-vivo activation on monocyte subsets remains to be studied. The first aim of the current study was to elucidate the effect of ex-vivo activation on activation marker expression of these cells in more detail.

Within the hospital the ex-vivo activation problem can, at least partially, be minimized by using a Point-of-Care automated flow cytometer. The use of such an automated flow cytometer greatly reduces analyses times to 15–20 min by eliminating time consuming manual sample preparation steps [5]. Also, by placing it Point-of-Care (i.e. near the patient) the time from venipuncture to start of analysis can be kept to a minimum.

Out-of-hospital flow cytometry analysis has invaluable (future) investigational and diagnostic potential in primary care, nursing homes and rural areas. However, minimizing ex-vivo activation of innate immune cells is even more challenging outside the hospital. The transportation of a blood sample collected in ambulatory care to the automated flow cytometer in the hospital takes time. This delay leads to ex-vivo activation of innate immune system, which hampers accurate interpretation of flow cytometry data. Fortunately, there is a potential strategy to minimize the ex-vivo activation bias of neutrophils, eosinophils and monocyte subsets by performing out-of-hospital, Point-of-Care automated flow cytometry analyses. The second aim of the current study was to investigate the feasibility and performance of the “bringing the lab to the sample” concept by placing a fully automated mobile flow cytometer into a van, thereby ‘bringing’ the analysis *to the patient* outside of the hospital.

## 2 Methods

### 2.1 Part one: Investigating ex-vivo activation of myeloid cells in a healthy cohort

**Fig. 1:**
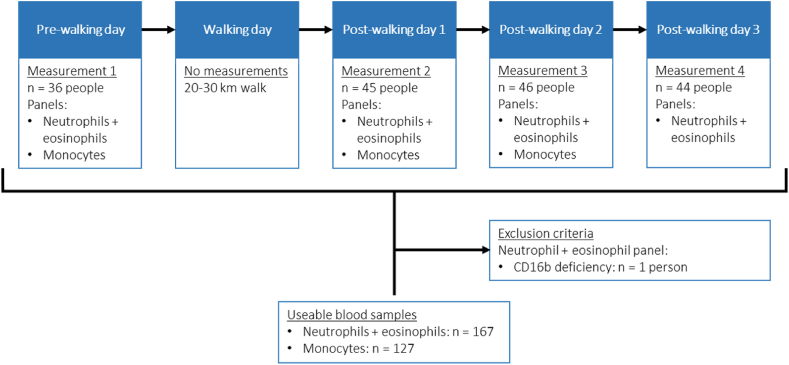
**Study flowchart for the healthy cohort that was used to study ex-vivo activation of neutrophils, eosinophils and monocytes.** Venous blood samples were collected from the cohort during several days around a 20–30 km walking exercise and were measured by an automated flow cytometer (see materials and methods section).

A fully automated flow cytometer (AQUIOS CL, Beckman Coulter, Indianapolis, IN, USA) was placed in a field laboratory. Point-of-Care flow cytometry analyses were performed on blood samples from a healthy cohort of 46 individuals [6]. A venous blood sample was collected in a sodium-heparin (Vacuette; Greiner Bio-One) blood collection tube on the day prior to and during 3 days after the day of prolonged walking exercise (20–30 km at a self-selected pace; Fig. 1). The AQUIOS CL was situated *stationary* in the study center next to the room where the venous blood samples were taken. Blood samples were stored at room temperature next to the machine and were analyzed with varying times between venipuncture and analysis. The time between venipuncture and analysis was accurately registered.

#### 2.1.1 Automated flow cytometry

Collected blood samples were automatically prepared and analyzed by the AQUIOS CL *Load & Go Flow Cytometer* (Beckman Coulter, Indianapolis, IN, USA). The operating methods of this machine have previously been described in detail [7]. In short, once the blood collection tube was placed in the machine, the machine fully automatically pipetted the blood simultaneously into two separate wells in a 96-deep well plate. To investigate cell responsiveness to an innate immune stimulus, the wells in the deep well plate were alternatingly uncoated/pre-coated with the activator N-Formyl-norleucyl-leucyl-phenylalanine (fNLF; end concentration 10^−5^ M; BioCat GmbH, Heidelberg, Germany). Next, the blood in the two separate wells was stained with a specific 5-marker antibody mix which was incubated for 15 min in the presence/absence of fNLF. Next, the red blood cells were lysed by adding a cyanide-free lytic (AQUIOS Lysing reagent A), which was stopped after 30 s by adding AQUIOS reagent B (both are from Beckman Coulter). This was directly followed by flow cytometric analysis.

#### 2.1.2 Antibody panels

The neutrophils, monocytes and eosinophils of every sample were analyzed with the use of two different antibody-panels. The antibody-panel for analyzing neutrophils and eosinophils contained CD16-FITC (clone 3G8), CD11b-PE (clone Bear1), CD62L-ECD (clone DREG56), CD10-PC5 (clone ALB1), CD64-PC7 (clone 22). The antibody-panel for analyzing monocytes contained CD11b-FITC (clone Bear1), CD169-PE (clone D21-075), CD16-ECD (clone 3G8), CD14-PC5 (clone RMO52), HLA-DR-PC7 (clone Immu-357). All antibodies were from Beckman Coulter (Marseille, France).

#### 2.1.3 Data of neutrophils, eosinophils and monocyte subsets

**Fig. 2:**
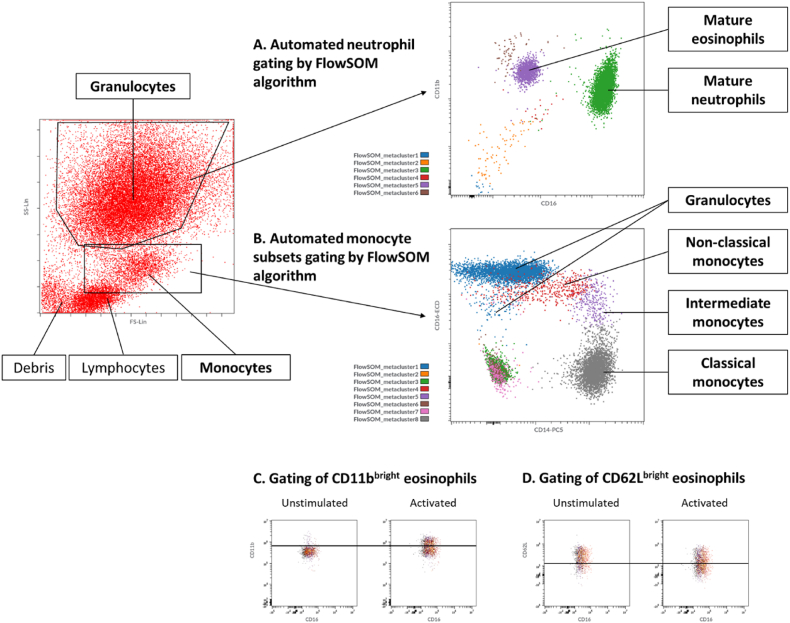
Automated gating of mature neutrophils (metacluster 3) and eosinophils (metacluster 5) by using the FlowSOM algorithm (64 clusters and 6 metaclusters) on samples measured with the antibody-panel for neutrophils and eosinophils (**A**). Automated gating of classical monocytes (metacluster 8), intermediate monocytes (metacluster 5), and nonclassical monocytes (metacluster 4) by the FlowSOM algorithm (100 clusters and 8 metaclusters) on samples measured with the monocyte antibody-panel (**B**). The percentage of CD11b^bright^ and CD62L^bright^ eosinophils was determined by using a predefined gate on the eosinophil cluster for CD11b and CD62L (**C** and **D**).

Flow cytometry standard (FCS) 3.1 High Res Listmode Files (.lmd) were exported form the device and imported into Cytobank (www.cytobank.org, a web-based flow cytometry analysis platform; Beckman Coulter, Indianapolis, IN, USA). Mature neutrophils and eosinophils were gated as previously described by Jukema et al., based on forward- and sideward scatter, followed by the FlowSOM algorithm (Fig. 2A) [7]. The percentage of CD11b^bright^ and CD62L^bright^ eosinophils was determined by using a predefined gate on the eosinophil cluster (Median Fluorescence Intensity (MFI) of 54 × 10^4^ for CD11b and MFI of 14 × 10^4^ for CD62L; Fig. 2C/D). Monocyte subsets were first gated based on forward- and sideward, followed by the FlowSOM algorithm (Fig. 2B), as previously described by Jukema et al. [8] The MFI's of the aforementioned markers of the different antibody-mixes were determined for the respective cell types. Each sample has two MFI values per marker: one for unstimulated cells (absence of fNLF) and one for activated cells (presence of fNLF; 10 μM). For every sample, the monocyte subset composition (percentage of classical, intermediate and non-classical) was assessed by dividing the event count of the different subsets by the event count of the total monocyte compartment.

### 2.2 Part two: Investigating the feasibility of a mobile flow cytometry laboratory

A *mobile* and fully automated AQUIOS CL was used for measuring blood collection samples during two out-of-the-hospital events: the TREAT 2022 study and PRO-COVID-19 study (see video 1 for illustration of the execution of the studies). Specific clinical outcome data with regard to the studies will be published elsewhere. This paper focusses solely on the feasibility data that were obtained during these studies.

#### 2.2.1 The *mobile* flow cytometry laboratory

The automated AQUIOS CL flow cytometer, was placed in the back of a van and securely stored in a travel case. Once the van arrived at the location of analysis the flow cytometer was accessed by partially removing the top of the travel case. Next, the wet-cart and operating personal computer were connected to an uninterruptible power supply (UPS). In turn, the UPS was connected to an external power supply. Flow cytometer start-up procedures were identical as performed in the hospital. The whole start-up procedure could be performed by a single person. The time from parking the van to being fully operational was recorded.

#### 2.2.2 Stability of the flow cytometry analysis

To check the stability of the flow cytometer measurements, two types of beads were analyzed at every location of analysis. Verification of the optical alignment and fluidics system was performed by measuring laser alignment verification beads (Flow Check™ Fluorospheres, Beckman Coulter, O'Callaghan's Mills, Ireland). Verification of the fluorescence detectors was performed by analyzing fluorescence verification beads (Flow-Set™ Pro Fluorospheres, Beckman Coulter, O'Callaghan's Mills, Ireland).

For the laser alignment verification beads, the AQUIOS CL determines a half peak coefficient of variation (HPCV), which was directly exported from the machine. The MFI's of the fluorescence verification beads were determined for every channel by importing the FCS files into Cytobank (www.cytobank.org, a web-based flow cytometry analysis platform; Beckman Coulter, Indianapolis, IN, USA). The laser alignment verification beads and the fluorescence verification beads were gated according to manufacturer recommendations.

#### 2.2.3 Statistical analysis

GraphPad Prism (version 8.3.0; Graphpad software, Inc., San Diego, CA, USA) was used to visualize the data and calculate statistics for all presented data. Linear regression analyses were performed for: 1) the MFI's of several (activation) markers on neutrophils, eosinophils and monocyte subsets versus the time till start of analysis; 2) the percentage of monocyte subsets versus the time till start of analysis; 3) the stability data of the beads (HPCV's and MFI's) versus the date of measurement.

## 3 Results

### 3.1 Part one: Investigating ex-vivo activation in a healthy cohort

Flow cytometric analyses were performed on blood samples from 46 healthy individuals. Due to logistical issues, blood samples from 10 people could not be analyzed on the pre-exercise day and the monocyte panel could not be analyzed on the third post-walking day. On the first and third day post-walking venipuncture was not performed for one and two participants, respectively. At the end of the study 167 blood samples were analyzed with the neutrophil and eosinophil panel and 127 blood samples were analyzed with the monocyte panel (Fig. 1).We varied the time between venipuncture and analysis to obtain an accurate analysis of the time dependency of the ex-vivo artificial activation. To investigate a broad spectrum of the kinetic profile of ex-vivo activation, the time between venipuncture and analysis by the machine for the entire study cohort ranged from 5 min to 8 h.

#### 3.1.1 Neutrophils activation in a blood collection tube starts from the moment the blood is drawn

**Fig. 3:**
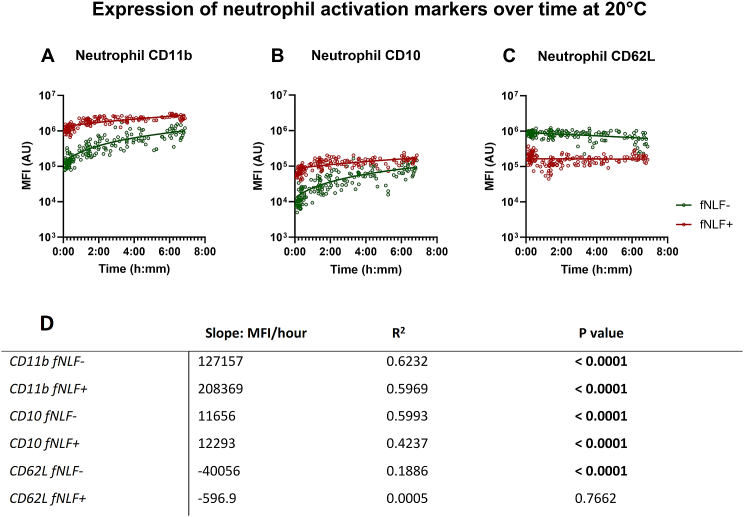
**Expression of neutrophil activation markers in regards to time till analysis on neutrophils obtained from a blood collection tube at room temperature.** Expression of CD11b, CD10 and CD62L is displayed for mature neutrophils (**A,B and C**). fNLF- samples were measured in the absence of fNLF, whereas fNLF+ samples were measured in the presence of the formylpeptide (10 μM). Linear regression analyses were performed for the median fluorescence intensity (MFI) in arbitrary units (AU) of the neutrophil activation markers with regard to the time between venipuncture and start of analysis by an automated flow cytometer. An F-test was used to determine whether the slope (time till analysis vs MFI) was significantly different from zero (**D**).

The expression of the activation markers CD11b (Mac-1) and CD10 (neprilysine/CALLA/neutral endopeptidase/enkephalinase) increased over time on unstimulated neutrophils present in the untouched blood sample (p < 0.0001 and p < 0.0001, respectively; Fig. 3A,B,D). On the other hand, CD62L expression decreased over time (p < 0.0001; Fig. 3C and D) which corroborated the activated phenotype of the neutrophils. CD11b and CD10 expression on fNLF-activated neutrophil also increased with extended time till analysis (p < 0.0001 and P < 0.0001, respectively; Fig. 3A,B,D). fNLF-activated neutrophil CD62L expression showed no negative relationship with time as the protein is instantly shed from the surface upon activation with formyl-peptides.

#### 3.1.2 Monocytes are susceptible for activation directly after blood collection, which alters their phenotypic characteristics

**Fig. 4:**
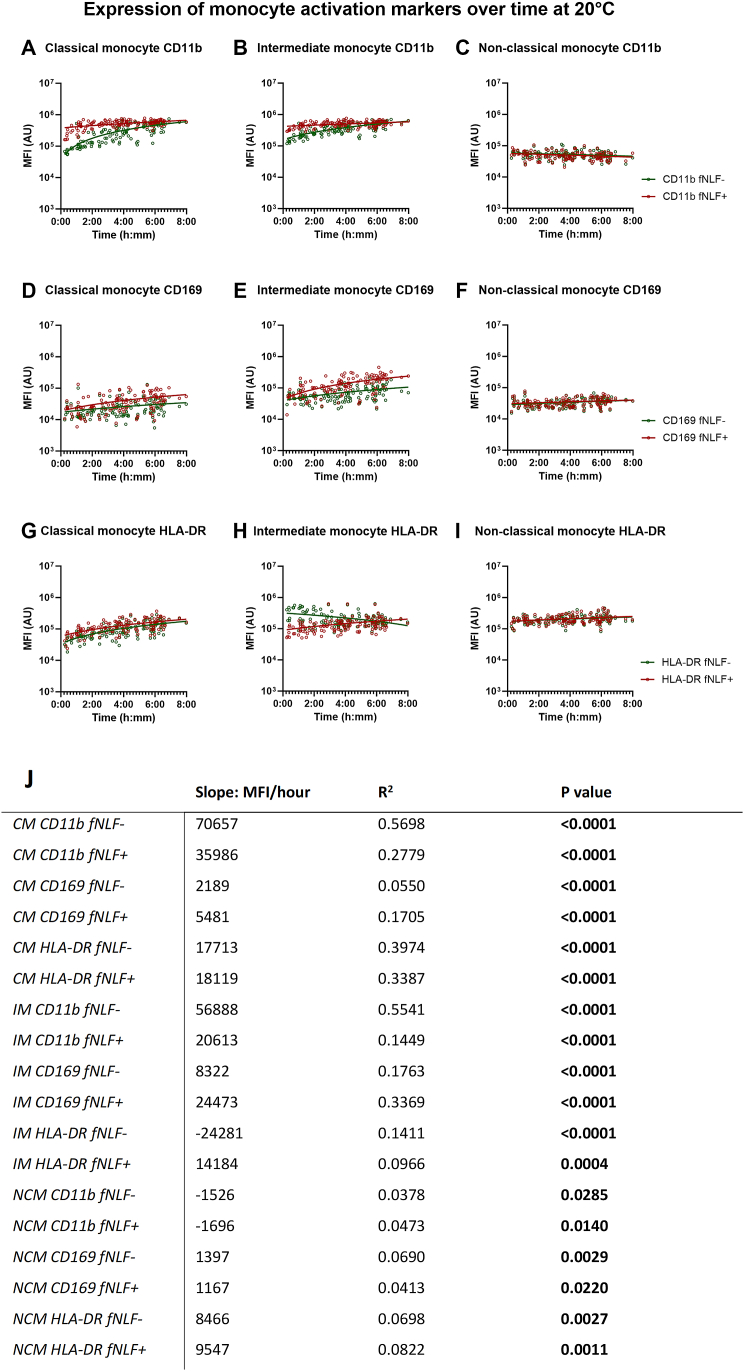
**Expression of monocyte** (**activation) markers in regards to time till analysis on monocytes obtained from a blood collection tube at room temperature.** Expression of CD11b, CD169 and HLA-DR is displayed for classical (**A,D and G**), intermediate (**B,E and H**) and non-classical monocytes (**C,F** and **I**). fNLF- samples were measured in the absence of fNLF, whereas fNLF+ samples were measured in the presence of the formylpeptide (10 μM).Linear regression analyses were performed for the median fluorescence intensity (MFI) in arbitrary units (AU) of the monocyte (activation) markers on monocyte subsets with regard to the time between venipuncture and start of analysis by an automated flow cytometer. An F-test was used to determine whether the slope (time till analysis vs MFI) was significantly different from zero (**J**). Classical monocyte (CM); Intermediate monocyte (IM); Non-classical monocyte (NCM).

CD11b, CD169 and HLA-DR expression on both unstimulated and fNLF-activated classical monocytes increased as the time till analysis increased (all p < 0.0001; Fig. 4A, D, G and J).

Unstimulated and fNLF-activated intermediate monocytes also showed a positive relationship between time till analysis and expression of CD11b (p < 0.0001 and p < 0.0001, respectively; Fig. 4B andJ) and CD169 (p < 0.0001 and p < 0.0001, respectively; Fig. 4E and J). Unstimulated intermediate monocytes showed a negative relationship between HLA-DR expression and time till analysis, whilst this was a positive relationship for fNLF-stimulated intermediate monocytes (p < 0.0001 and p = 0.0004, respectively; Fig. 4H andJ).

Unstimulated and fNLF-stimulated CD11b expression on non-classical monocytes showed a negative relationship with time till analysis (p = 0.0285 and p = 0.0140, respectively; Fig. 4C andJ) albeit the extent of the decrease was limited. Unstimulated non-classical monocytes showed increased expression CD169 and HLA-DR as time till analysis increased (p = 0.0029 and p = 0.0027, respectively; but again the effect was moderate (Fig. 4 F, I andJ). fNLF-activated non-classical monocytes also showed a positive relationship between CD169 and HLA-DR expression and time till analysis (p = 0.0220, p = 0.0011, respectively; Fig. 4F, I andJ).

#### 3.1.3 Monocyte subset composition changes over time, but is also altered with fNLF-activation

**Fig. 5:**
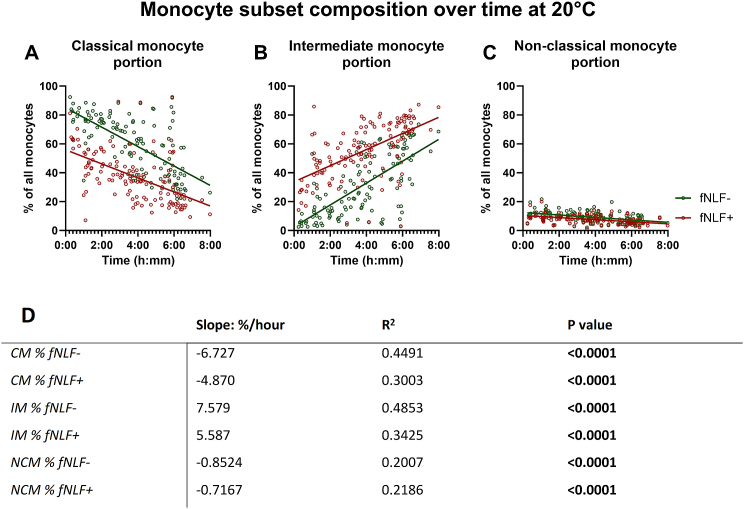
**Composition of monocyte subsets in regards to time till analysis of monocytes obtained from a blood collection tube at room temperature.** The portion of the classical (**A**), intermediate (**B**) and non-classical monocytes (**C**) is displayed. fNLF- samples were measured in the absence of fNLF, whereas fNLF+ samples were measured in the presence of the formylpeptide (10 μM). Linear regression analyses were performed for the percentage of monocyte subsets of all monocytes with regard to the time between venipuncture and start of analysis by an automated flow cytometer. An F-test was used to determine whether the slope (time till analysis vs percentage of monocyte subset) was significantly different from zero (**D**). Classical monocyte (CM); Intermediate monocyte (IM); Non-classical monocyte (NCM).

The composition of the monocyte subsets also changed over time in the blood collection tube. As time till analysis increased, the classical monocyte portion decreased in both unstimulated and fNLF-stimulated samples (p < 0.0001 and P < 0.0001, respectively; Fig. 5A andD), whilst the intermediate monocyte portion seemed to increase in both sample types (p < 0.0001 and P < 0.0001, respectively; Fig. 5B andD). Moreover, the portion of non-classical monocytes decreased in both unstimulated and fNLF-stimulated samples as the time till analysis increased (p < 0.0001 and P < 0.0001, respectively; Fig. 5C and D).

Paired analysis of unstimulated and fNLF-activated samples revealed that fNLF-activation decreased the classical monocyte portion, whilst the intermediate monocyte portion increased (p < 0.0001 and p < 0.0001, respectively; Fig. 5A and B). Non-classical monocytes showed a decrease after fNLF-activation (p < 0.0001; Fig. 5C).

#### 3.1.4 Eosinophils are slightly sensitive for activation in a blood collection tube

**Fig. 6:**
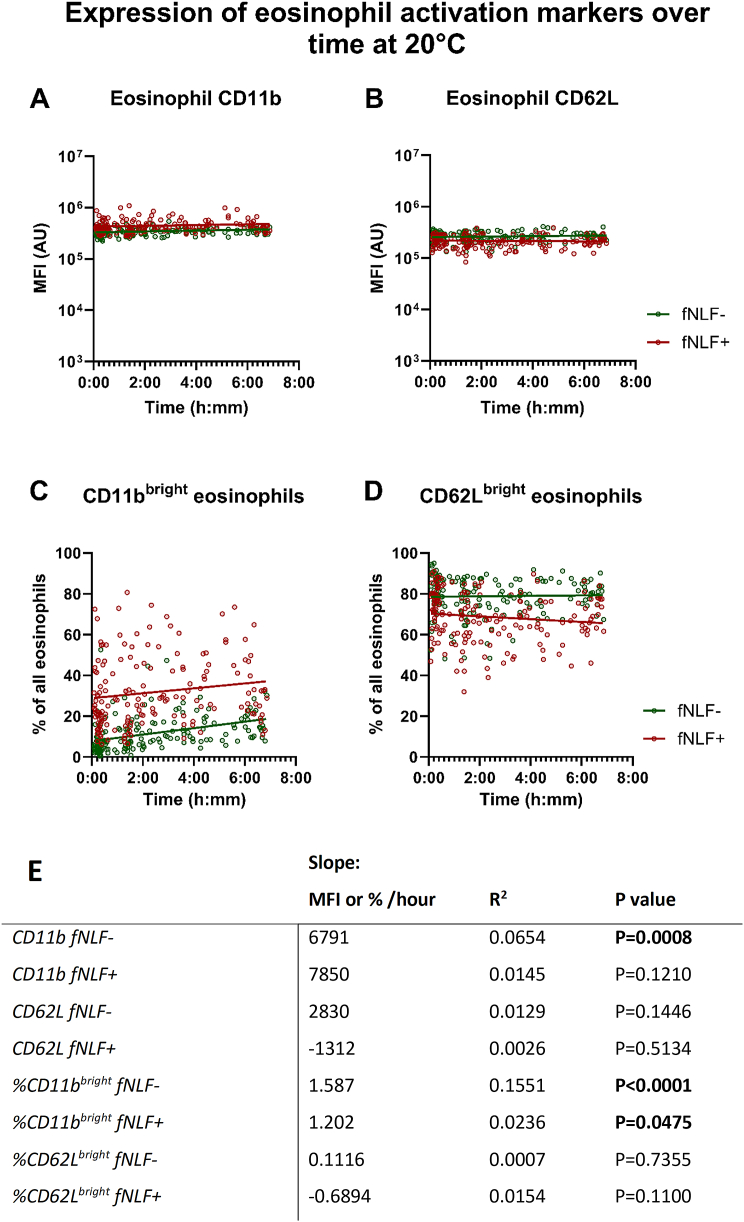
**Expression of eosinophil activation markers in regards to time till analysis on eosinophils obtained from a blood collection tube at room temperature.** Expression of CD11b and CD62L (**A** and **B**) and the percentage of CD11b^bright^ and CD62L^bright^ cells (**C** and **D**) is displayed for mature eosinophils. fNLF- samples were measured in the absence of fNLF, whereas fNLF+ samples were measured in the presence of the formylpeptide (10 μM). Linear regression analyses were performed for the time between venipuncture and start of analysis by an automated flow cytometer with regard to 1) the median fluorescence intensity (MFI) in arbitrary units (AU) of the eosinophil activation markers and 2) the percentage of CD11b^bright^ and CD62L^bright^ eosinophils. An F-test was used to determine whether the slope (time till analysis vs MFI or percentage of bright cells) was significantly different from zero (**E**).

The CD11b expression and the percentage of CD11b^bright^ cells on unstimulated eosinophils showed a positive relationship with regard to time till analysis (p = 0.0008 and p < 0.0001, respectively; Fig. 6A, C and E). fNLF-stimulated samples showed a positive relationship between the percentage CD11b^bright^ eosinophils and time till analysis (p = 0.0475; Fig. 6B and E). Compared to the other cell types, the slopes of the relationships for eosinophils between time of venipuncture and analysis were substantially less pronounced/steep.

### 3.2 Part two: investigating the feasibility of a mobile flow cytometry laboratory

**Fig. 7:**
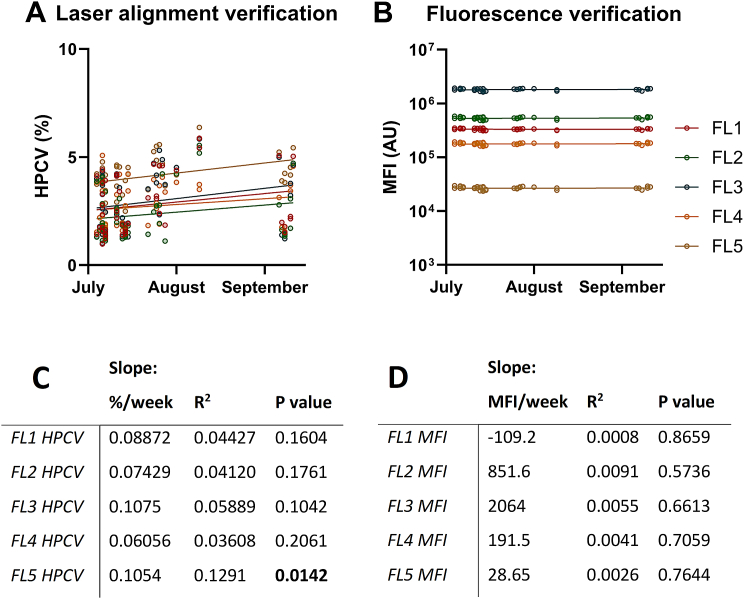
**Testing of the stability of the flowcytometric analysis was performed by the measurement of stability beads in the mobile flow cytometry laboratory over the course of 3000 km.** The half peak coefficient of variation (HPCV) of laser verification beads and the median fluorescence intensity (MFI) in arbitrary units (AU) of fluorescence verification beads are displayed (**A** and **B**). Linear regression analyses were performed for 1) the for the HPCV and the MFI values with regard to timing of the analysis during 3000 km of driving. An F-test was used to determine whether the slope (timing of analysis vs HPCV or MFI) was significantly different from zero (**C** and **D**).

The “bringing the lab to the sample” concept was accomplished by travelling over 3000 km with the mobile flow cytometry laboratory: a total of 144 blood samples were analyzed for two other studies at 19 locations between July 4th and September 11th, 2022. See video 1 for an impression on the implementation of the mobile flow cytometry laboratory during these studies. Whilst performing measurements for these studies the half peak coefficient of variation of the alignments beads for fluorescence channel 5 had a positive relationship with distance travelled (p = 0.0142; Fig. 7A and C), whilst no such relationship was found for the other channels. Nonetheless, the measured MFI's of the fluorescence verification beads also showed no relationship with distance travelled (Fig. 7B andD).

Supplementary data related to this article can be found online at https://doi.org/10.1016/j.heliyon.2024.e28883

The following are the Supplementary data related to this article:

Video 1 Implementation of the mobile flow cytometry laboratory during two different studies. Video 1

#### 3.2.1 The mobile flow cytometry laboratory has a short setup time

**Fig. 8:**
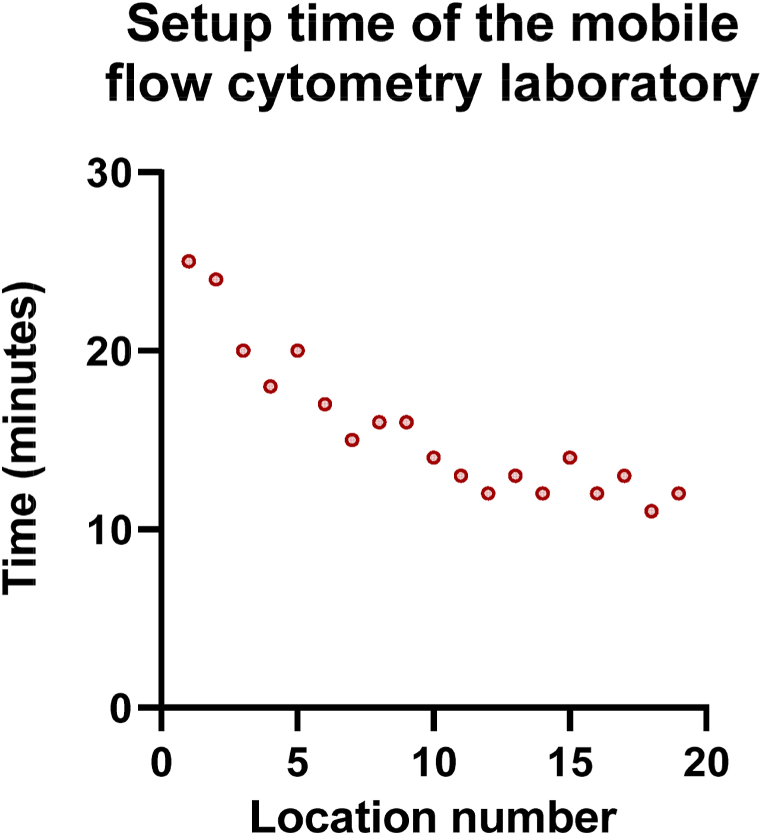
**The setup time of the mobile flow cytometry laboratory with regard to different setup locations.** The time till analysis was measured from the moment of parking the van till the flow cytometry laboratory was fully operational for analyses.

Overall, the median time from parking the mobile flow cytometry laboratory to being fully operational, which was performed by a single person, was 14 min (IQR 12–18 min). However, the setup time decreased as the number of locations increased (Fig. 8). In the last 5 stops, the median setup time decreased to 12 min (IQR 11,5–13,5 min).

## 4 Discussion

This study had two aims. First, the extent and kinetics of ex-vivo activation of neutrophils, eosinophils and monocyte subsets present in a blood collection tube obtained from a cohort of healthy volunteers was investigated. We found that ex-vivo activation of these innate effector cells starts from the moment the blood is drawn. Second, this study investigated the feasibility of a mobile flow cytometry laboratory. We clearly showed that out-of-hospital Point-of-Care flow cytometry analysis is feasible. Combined these data indicate that the “bringing the lab to the sample” approach is necessary for accurately interpreting flow data related to the innate immune system's state during out-of-hospital diagnostics for infections and inflammatory conditions. This strategy ensures correct interpretation of the innate immune response by minimizing ex-vivo activation bias. Elaborating on previous research, this approach can be useful for numerous settings and conditions, i.e. from fast infection diagnostics in primary care to immune monitoring of tissue damage in athletes during training or sporting events.

Different types of inflammation require attuned flow analysis as illustrated by the fact that DAMP induced inflammation is particularly driven by neutrophil inflammation whereas T2-type inflammation is more dominated by eosinophils [[9], [10], [11], [12]]. The different types of innate immune cells are differentially sensitive for ex-vivo manipulation and, therefore will be discussed separately.

### 4.1 The neutrophil compartment is very sensitive for ex-vivo manipulation

As shown in Fig. 3 neutrophils quickly acquire an activated phenotype post venipuncture. This phenotype is illustrated by the increased expression of the activation markers Mac1 (CD11b) and neprilysin (CD10) and the down regulation of L-selectin (CD62L) [5,7,13]. This was also apparent for expression of CD11b and CD10 expression on fNLF-activated neutrophils, which indicates that the artificial ex-vivo environment both directly activated the cells and primed the cells for activation by formylpeptides. CD62L expression on fNLF-activated neutrophils showed no relationship with time between venipuncture and analysis. This was not surprising as the used concentration of fNLF already fully induced the ‘shedding’ of CD62L, leaving no opportunity for the priming of even more downregulation induced by time dependent ex-vivo activation. These data imply that most studies on primed and/or activated neutrophils in vivo should be reevaluated as the increase of activation markers in vitro might not be happening in vivo, but is a result of enhanced sensitivity for ex-vivo manipulation. It must be emphasized that changes in fNLF-induced activation of innate immune cells are poorly understood in terms of mechanisms, but they can be mediated, for instance, by a change in expression of formyl peptide receptors [14].

### 4.2 Ex-vivo activation compromises the accurate determination of the composition of monocyte subsets and their activation status

The situation with ex-vivo activation of the monocyte compartment is even more complex as the time to analysis is associated with both a change in expression of activation markers, and with a shift from classical (CD14^bright^/CD16^dim^) to intermediate monocytes (CD14^bright^/CD16^bright^). This conclusion is supported by the finding that the expression of the activation markers (CD11b and CD169) increased in a time dependent manner on both unstimulated and fNLF-activated monocytes. This is in line with previous research that showed that standard sample work-up procedures upregulated monocyte activation markers [15].

Counterintuitively, HLA-DR expression on unstimulated intermediate monocytes decreased as time till analysis increased. This can be explained by the fact that all monocyte subsets showed a positive relationship between unstimulated CD16 expression and time till analysis (Fig. S1). This leads to a confusing situation as CD16 is also used to distinguish classical monocytes (CD16^dim^/CD14^bright^) from intermediate monocytes (CD16^bright^/CD14^bright^) and non-classical monocytes (CD14^dim^/CD16^bright^). The increased expression of CD16 over time on classical monocytes causes these cells to become misclassified as intermediate. This makes the interpretation of these findings complex as it seems that (CD14^bright^/CD16^bright^) intermediate monocytes consists of two populations: (in vitro) activated classical cells and true intermediate monocytes. This view is supported by the finding that the relative decrease in classical monocytes is similar to the relative increase in intermediate monocytes, both non-stimulated and in the presence of fNLF. This hypothesis is reinforced by a study that found that, compared to whole blood analysis, gradient purification by ficoll leads to increased CD16 expression on classical monocytes and a decreased number of CD14^bright^/CD16^dim^ monocytes [16].

Our data has important implications for the interpretation of markers such as HLA-DR on monocytes. Classical monocytes have a lower HLA-DR expression than intermediate monocytes [16,17]. The artificial shift from classical to intermediate cells based on increased CD16 expression brings HLA-DR^dim^ cells to the population of intermediate cells. As time progresses more HLA-DR^dim^ classical monocytes get artificially misclassified as intermediate monocytes, seemingly lowering the median HLA-DR signal of the intermediate monocyte subset. This complicates conclusions based on data regarding intermediate monocytes.

(Un)stimulated non-classical monocytes (CD16^bright^/CD14^dim^) also showed alterations in marker expression upon prolonged time to analysis, but these were not as pronounced as the situation with intermediate and classical monocytes. The relative number of non-classical monocytes decreased as time till analysis increased and was also slightly lower after fNLF-activation. We do not have an explanation for this finding, but non-classical monocytes have been described to easily adhere to surfaces or other cells. So, this behavior might be attributed to a small loss of the number of these cell by adhesion processes [3].

Together, these data underline the susceptibility of monocytes to artificial ex-vivo activation. The interpretation of data showing increased intermediate monocyte counts in for instance inflammatory and autoimmune conditions should be reevaluated based on the possibility that part of the increase of intermediate monocytes can be explained by artificial increase of expression of CD16 during these inflammatory conditions [[18], [19], [20]]. Moreover, HLA-DR is a marker for disease severity and outcome in infections and sepsis associated immunoparalysis [[21], [22], [23], [24]]. Our findings indicate that HLA-DR gets artificially upregulated as a result of ex-vivo activation. Potentially, this also happens in patients with immunoparalysis, potentially leading to wrong conclusions.

### 4.3 Eosinophils are only slightly sensitive to ex-vivo activation

The eosinophil compartment seems least sensitive for ex-vivo manipulation as (un)stimulated eosinophils only showed minimal time till analysis dependent alterations in activation marker expression. As eosinophils have evolved as important cells in combating parasite infections they employ their cytotoxic potential extracellularly as the target is much bigger than the cells themselves [4]. It is tempting to speculate that activation of this highly cytotoxic cell type should be very well controlled to prevent unwanted tissue damage such as seen in tissues affected by unbalanced Th2-responses [25]. To strengthen this view we have shown that eosinophils only respond to opsonized targets after priming by cytokines or bioactive lipids. This priming behavior acts as an extra lock for cellular activation [26]. The absence of short term ex-vivo activation of eosinophils makes them an interesting target for reliably studying and/or diagnosing T2-inflammation [27].

### 4.4 The “bringing the laboratory to the sample” circumvents out-of-hospital ex-vivo activation

Our data illustrate that all studied innate immune cell types, but particularly neutrophils and classical monocytes, show time till analysis dependent alterations in expression of activation markers. This phenomenon is not limited to innate effector cells. Changes in surface marker expression after venipuncture have also been described for lymphocyte subsets. To bridge the gap between out-of-hospital collection and laboratory flow cytometry analyses, fixation of lymphocytes has been studied [28]. However, fixation procedures can introduce artefacts on immune cells in samples from healthy and ill donors, which can best be avoided by analyzing live whole blood [29]. Also, different whole blood stabilizers give variable results with respect to various leukocyte populations [30].

This emphasizes the importance of quick and fully standardized analysis of these cells to prevent misinterpretation of flow cytometry data. Fully automated Point-of-Care flow cytometry meets these requirements and now can be applied both within and outside the hospital. This brings inflammation research to a next level, which is not limited to analyzing innate effector cells, but also applies to lymphocytes and other cells that are susceptible for ex-vivo activation. Also, the logistical hurdles of facilitating a mobile flow cytometry laboratory are similar to manipulating out-of-hospital samples for subsequent analysis, which also requires a small field laboratory on the site of sample collection. Up till now classical flow cytometry in the diagnostics of inflammatory and infectious conditions has not been deployed to its full potential, because of the timing issues described in this study. This new mobile flow cytometry approach opens up new possibilities on, for instance, out-of-hospital bedside infections diagnostics, which has already shown its Point-of-Care potential inside hospital walls [8]. It is also useful for investigating the immune response to physiological stress, such as evoked by endurance exercise, at remote locations [31].

Surely, optimal laser alignment is crucial for reproducible flow cytometry measurements in a van outside the hospital setting [32]. Our data demonstrate the stability and reproducibility of the measurements. Also, the robustness of the set-up was illustrated by the stable laser alignment and fluorescence sensitivities with very low variation coefficients in the different channels. Moreover, the setup time of the mobile flow cytometry laboratory also greatly determines the feasibility of the concept. In this study, the median setup time by an individual person was 14 min and it showed a decreasing trend over time.

### 4.5 Strengths and limitations

To our knowledge, this is the first extensive study in whole blood that demonstrates the time dependent ex-vivo activation of effector cells of the innate immune system after regular blood collection. Combining automated flow and semi-automated FlowSOM based gating minimized human error and bias from the manual gating and analysis process, increasing the quality of the presented data. Also, this is the first study to investigate the feasibility and performance of automated out-of-hospital Point-of-Care flow cytometry analysis. Our study also has some limitations. First, the blood samples of individuals were not measured repetitively. The relationship between activation data and time till analysis were based on combining the different samples of different participants with different times till analysis, instead of measuring a single blood collection tube at different timepoints. However, measuring a single tube multiple times over the day would drastically decrease the number of different donors that could be measured each day. Nonetheless, our previous initial data on a small subset of donors that were measured repetitively lead to the same conclusion that prolonged time till analysis leads to activation in the blood collection tube [5]. Second, the analysis by the AQUIOS is based on a no-wash procedure and fast lysis of red blood cells. This set-up is different from most classical flow procedures and, therefore, might be difficult to compare. Also, here we only assessed changes in blood collection tubes with sodium-heparin as anticoagulant. Potentially, ex-vivo activation might be less pronounced in blood collection tubes with Ca^2+^-binding based anticoagulation. However, assessment of neutrophil responsiveness to fNLF is hampered in Ca^2+^-based anticoagulated blood, as neutrophil activation is at least partially mediated by Ca^2+^-signaling [33]. Lastly, we exported the flow cytometry data from the flow cytometer and analyzed these on an external platform to perform semi-automated clustering strategies. This was done to perform the automated clustering (FlowSOM) based gating that should be implemented to the optimize flow cytometry based identification of cells.

## 5 Conclusion and implications for future practice

This study found that ex-vivo activation of innate immune effector cells starts to occur immediately after venipuncture. This study also revealed that mobile, fast, automated out-of-hospital Point-of-Care flow cytometry is a feasible approach that bypasses most of the technical hurdles in cellular diagnostics of inflammation and infection associated processes. This allows for fast and reliable diagnostics that can be applied Point-of-Care and in a fast and robust way. Also, this approach allows the assessment off potential differences in cell reactivity induced by formyl-peptides, that might be linked with changes in intracellular signaling in innate immune cells in certain disease conditions. In conclusion, the mobile flow cytometry laboratory approach fits in a future scenario where ‘simple’ flow-based diagnostics will be available outside hospital walls.

## Ethics statement

This study conformed to the principles of the Declaration of Helsinki, was approved by the Medical Ethical Committee of the 10.13039/501100006209Radboud University Medical Center (Study-ID: NL77522.091.21) and was registered at the Dutch trial registry (#NL9499). All participants provided written informed consent to participate in the study prior to any experimental procedure.

## Funding

Nothing to declare.

## Data availability statement

The data will be made available on reasonable request.

## CRediT authorship contribution statement

**B.N. Jukema:** Writing – review & editing, Writing – original draft, Visualization, Methodology, Investigation, Formal analysis. **T.C. Pelgrim:** Writing – review & editing, Methodology. **M. Spoelder:** Writing – review & editing, Methodology. **C.C.W.G. Bongers:** Writing – review & editing, Methodology, Conceptualization. **M.T.E. Hopman:** Writing – review & editing, Methodology, Conceptualization. **K. Smit:** Writing – review & editing, Methodology. **M.H. Rijk:** Writing – review & editing, Methodology. **R.P. Venekamp:** Writing – review & editing, Conceptualization. **N. Vrisekoop:** Writing – review & editing, Conceptualization. **L. Koenderman:** Writing – review & editing, Writing – original draft, Supervision, Methodology, Conceptualization.

## Declaration of competing interest

The authors declare the following financial interests/personal relationships which may be considered as potential competing interests:

B.N. Jukema reports equipment, drugs, or supplies was provided by Beckman Coulter Inc. The authors declare that the research was conducted in the absence of any commercial or financial relationships that could be construed as a potential conflict of interest. If there are other authors, they declare that they have no known competing financial interests or personal relationships that could have appeared to influence the work reported in this paper.
