# Supplementary material for: Automated, Point-of-Care mobile flow cytometry: Bringing the laboratory to the sample
Source: Heliyon. 2024 Apr 3;10(8):e28883. doi: 10.1016/j.heliyon.2024.e28883 (PMC11019183; doi:10.1016/j.heliyon.2024.e28883)
Supplement: Multimedia component 1 [file mmc1.docx]

**Supplementary figures**

| D | Slope:  MFI/hour | R^2^ | P value |
| --- | --- | --- | --- |
| CM CD16 fNLF- | 95.4 | 0.1578 | **<0.0001** |
| CM CD16 fNLF+ | 134.8 | 0.0162 | 0.1536 |
| IM CD16 fNLF- | 6873 | 0.3385 | **<0.0001** |
| IM CD16 fNLF+ | 6989 | 0.3308 | **<0.0001** |
| NCM CD16 fNLF- | 2716 | 0.0568 | **0.0070** |
| NCM CD16 fNLF+ | 2446 | 0.0231 | 0.0882 |

**Figure S1. Expression of CD16 on monocyte subsets in regards to time till analysis on monocytes** **obtained from a blood collection tube at room temperature.** Expression of CD16 is displayed for classical (**A**), intermediate (**B**) and non-classical monocytes (**C**). fNLF- samples were measured in the absence of fNLF, whereas fNLF+ samples were measured in the presence of the formylpeptide (10 μM). Linear regression analyses were performed for the median fluorescence intensity (MFI) in arbitrary units (AU) of monocyte CD16 on several monocyte subsets with regard to the time between venipuncture and start of analysis by an automated flow cytometer. An F-test was used to determine whether the slope (time till analysis vs MFI) was significantly different from zero (**D**). Classical monocyte (CM); Intermediate monocyte (IM); Non-classical monocyte (NCM).

**Video 1: Implementation of the mobile flow cytometry laboratory during two different studies.**
